# Investigating criteria for valuation of forage resources by local agro-pastoralists in West Africa: using quantitative ethnoecological approach

**DOI:** 10.1186/s13002-018-0261-4

**Published:** 2018-10-23

**Authors:** John-Baptist S N Naah

**Affiliations:** 1 Center for Development Research (ZEF), University of Bonn, Walter-Flex 3, 53113 Bonn, Germany; 2 Geographical Institute, University of Cologne, Otto-Fischer Straße 4, 50674 Cologne, Germany

**Keywords:** Agro-pastoralists, Burkina Faso, Forage plants, Ghana, Local valuation criteria, Social-ecological systems

## Abstract

**Background:**

This paper provides an insightful quantitative ethnoecological analysis and affirms that agro-pastoralists have a multiplicity of criteria for valuating their natural forage resources. Rural households in West Africa are not only confronted with water resource scarcity but also have to cope with limited forage resources to feed livestock in both wet and dry seasons based on local knowledge. Local agro-pastoral social-ecological systems (SESs) in the study areas stem from the daily utilization of available forage resources by dominant domestic livestock (cattle, goats, and sheep) over the years. However, there is very little systematic knowledge documentation on forage-related valuation criteria in this part of the world. Hence, this study aimed at examining (1) forage resources used for different seasons and livestock types, (2) explicit forage-related valuation criteria and associated salience, and (3) effects of socio-demographic and climatic aridity on local valuation criteria.

**Methods:**

To address these aims, a total of 526 individual ethnoecological interviews (encompassing Dagbani, Gurunsi, and Mossi ethnic groups) were conducted in 16 villages coupled with vegetation sampling of 144 plots in Ghana and Burkina Faso. Rigorous model selection, generalized linear mixed-effects models, cognitive salience indices, and descriptive statistics were applied.

**Results:**

The results revealed that majority (73%) of the agro-pastoralists regarded herbaceous forage plants to be very palatable for livestock consumption in the rainy season and for cattle while woody vegetation and crop-related forage plants were rather perceived to be more important in the dry season and for goats and sheep. The findings also indicated that climatic aridity significantly influenced the number of forage-related valuation criteria cited by agro-pastoralists for different seasonal and livestock types (*p* < 0.001). It was also found out that agro-pastoralists did not only judge forage plants based on their availability but also on other criteria such as palatability, stimulation of milk production, and healthy growth of livestock.

**Conclusion:**

Local agro-pastoralists’ knowledge on natural forage resources and their valuation criteria is geared towards sustainable domestic livestock production. This study is thus interesting and crucially important for fellow scientists, policy-makers, and other stakeholders in the agricultural production sector in local farming landscapes within West Africa and beyond.

## Background

The world’s drylands constitute approximately 41.3% of the terrestrial landmass of our planet [1], which support more than two billion people (one third of humanity) and 90% of whom live in developing countries [2]. These global drylands have expanded in the last six decades and will continue to expand in this twenty-first century under a warming climate [3]. Such expansion of global drylands will negatively affect many people, especially rural farmers [3] and lead to decline in natural forage resources for livestock grazing. The main source of livelihood for about 1.3 billion smallholder farmers worldwide is said to be agriculture, which is particularly susceptible to impacts of climate change [4]. Being well-known as the backbone of the West African economy, the agricultural sector employs over 50% of the labor force in Ghana [5] and about 80% of the economically active population in Burkina Faso [6].

The West African Sub-Sahara (WASS) is characterized by a semi-arid climate with a high rainfall variability and regarded as one of the poorest regions in the world [7]. The high degree of inter- and intra-annual rainfall variability in this region not only causes highly variable forage quality and quantity but also seriously limits other ecosystem provisioning services [1, 8, 9] and therefore aggravates the living conditions of the vulnerable rural poor [10].

In spite of climate-related risks and human-induced impacts on the rural populations, local farmers persistently cope with such challenges and still forge ahead to meet their daily basic needs of life [11]. For instance, local pastoralists in the semi-arid region of Morocco have been reported of using their “old strategies” to adapt to the new, changing climate [12] for their survival. Across semi-arid environments in WASS, several studies have either used local ecological knowledge (LEK) approach to investigate savanna trees including their use value and management [13–18] or analyzed pastoral management patterns in the West African region [19, 20]. Over the years, humans (e.g., local agro-pastoralists) and nature (e.g., forage species) have co-existed, leading to development of adaptive and complex social-ecological systems—SESs [21]. These adaptive and intricate (agro-)pastoralists’ SESs are hinged on extensive utilization of available natural forage plants for their livestock-based livelihoods and culture [22].

According to the ecological apparency hypothesis [23, 24], the apparent plants are commonly used and highly valued by beneficiary users as compared to the fewer and smaller ones. Literature has also shown that elaborate LEK studies on different forage plants, including their palatability, phenology, life history and availability on local pastures, are of crucial importance [25–27]. Thus, the valuation of forage resources by local land users is a crucially important component of an adaptive natural resource management [28]. Specifically, in northern Ghana and southern-central Burkina Faso, very recent studies in the same study area focused on spectral indicators of forage quality [29], factors influencing the distribution of local ecological knowledge of forage resources [30], and environmental drivers of forage provision and erosion control [31]. Notwithstanding, these studies failed to address the aspect of local valuation criteria for available forage resources from the perspectives of local agro-pastoralists in the West African Sudanian savannas. Moreover, little is still known about how socio-demographic and climatic variables influence local agro-pastoralists’ decisions on natural forage resources utilization for sustainable livestock production in such vastly under-documented part of the world. This study did not only focus on studying local valuation criteria for herbaceous forage plants (grasses and forbs) and woody but also considered crop-related forage plants used by domesticated livestock (e.g., cattle, goats, and sheep). This is because these livestock types have varied feeding preferences. It was hypothesized that local valuation of forage resources is based on several criteria during different seasons (wet and dry) and for different livestock types namely cattle (*Bos taurus* L.), goats (*Capra hircus* L.), and sheep (*Ovis aries* L.). It is estimated that about 25% of cattle, 33% of sheep, and 40% of goats are reared among smallholder farmers in WASS [32]. The specific breeds of cattle reared in northern Ghana and southern Burkina Faso include the West African Shorthorn (humpless type), Zebu/White Fulani, and crossbreed such as Sanga and N’Dama [33]. These breeds of cattle are reared in the study areas because they are trypanotolerant and therefore able to adapt to tsetsefly-infested environments [33]. For sheep and goats, the common breeds reared by local farmers include the West African long-legged type (mostly found in Ghana), Djallonke, and crossbreed Mossi type (common in Burkina Faso) [34]. It was also presumed that identifying plants or groups of plants that are judged as important by local people can effectively assist the conservation and management of keystone natural forage plants and thus ensure the reproductive success of livestock. The major objectives of this study are:

1. To find out forage types crucially relevant for livestock consumption in different seasonal contexts.
2. To identify local criteria for valuation of forage plants among agro-pastoralists and to assess their salience for livestock production.
3. To investigate how the socio-demographic and climatic aridity variables affect the citation of local valuation criteria for forage resources.

## Methods

### Environmental setting

**Fig. 1:**
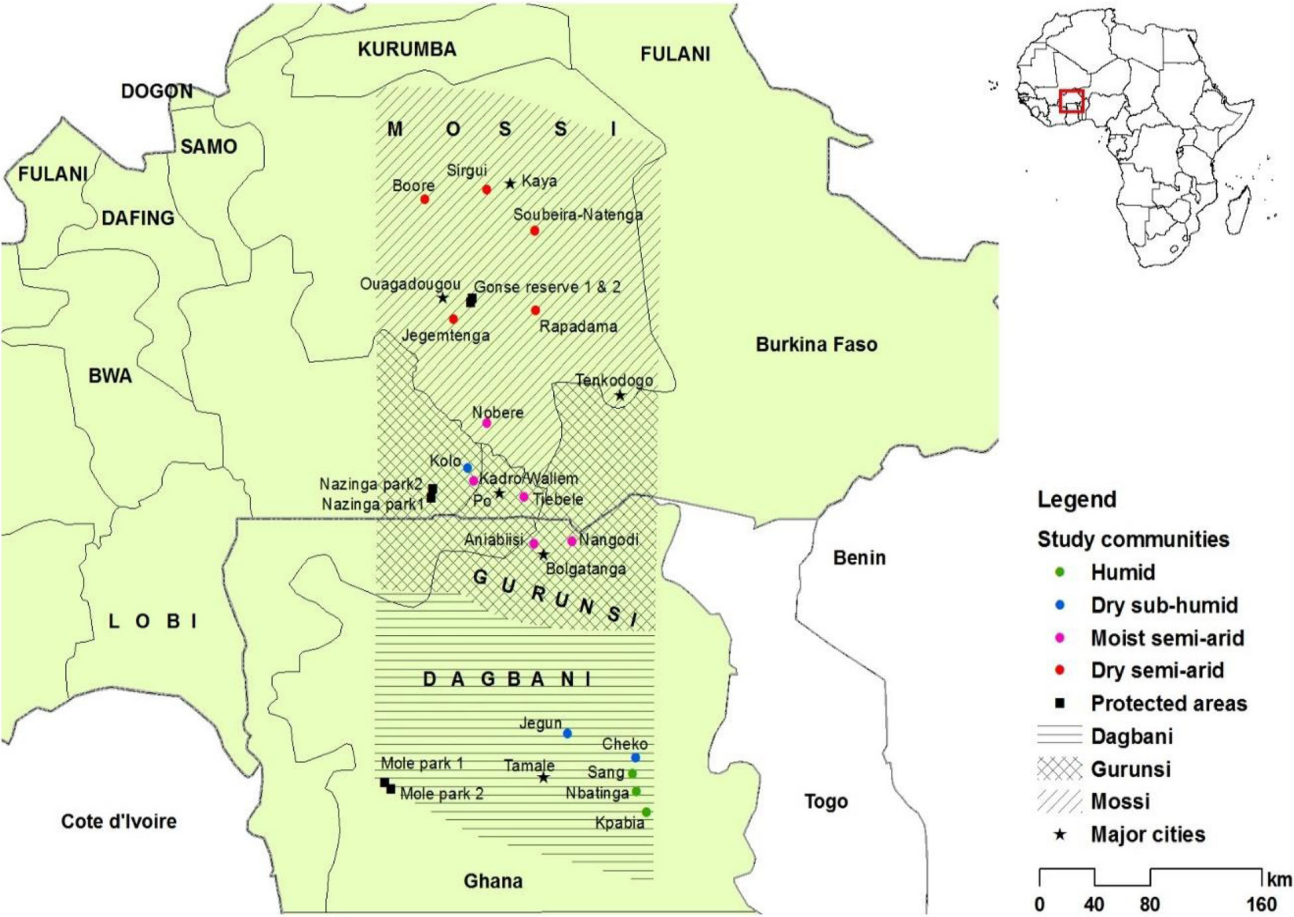
Map depicting the three major ethnic groups in the study area and the climatic aridity classes located within northern Ghana and southern-central Burkina Faso

The ethnoecological surveys among local agro-pastoralists encompassed northern Ghana and southern-central Burkina Faso. This wider study area covers about 530 × 200 km^2^ north-south extension [29–31], representing a steep climatic aridity gradient within the West Africa’s Guinean and Sudanian savannas (Fig. 1). Thus, the southern part of the study area covers the Ghana side with dry sub-humid conditions to humid while the northern portion of it encompasses Burkina Faso with harsher, drier semi-arid weather conditions. The intermediate aridity class (moist semi-arid) lies in-between, making four climatic aridity classes delineated for this study. The climate of the study area is characterized by a unimodal rainy season starting from April to October in the south and around May to August in the north. The mean annual precipitation (MAP) in the southernmost part ranges between 800 and 1500 mm [35]. The MAP for the intermediate climatic zone declines to about 700 to 1200 mm [36] and then further falls to about 750 to 950 mm in the northern part of the study area in Burkina Faso [37]. Farming activities are predominantly undertaken by local agro-pastoralists in the rainy season. The harmattan period (dry season) begins in December and ends in March in both countries.

**Fig. 2:**
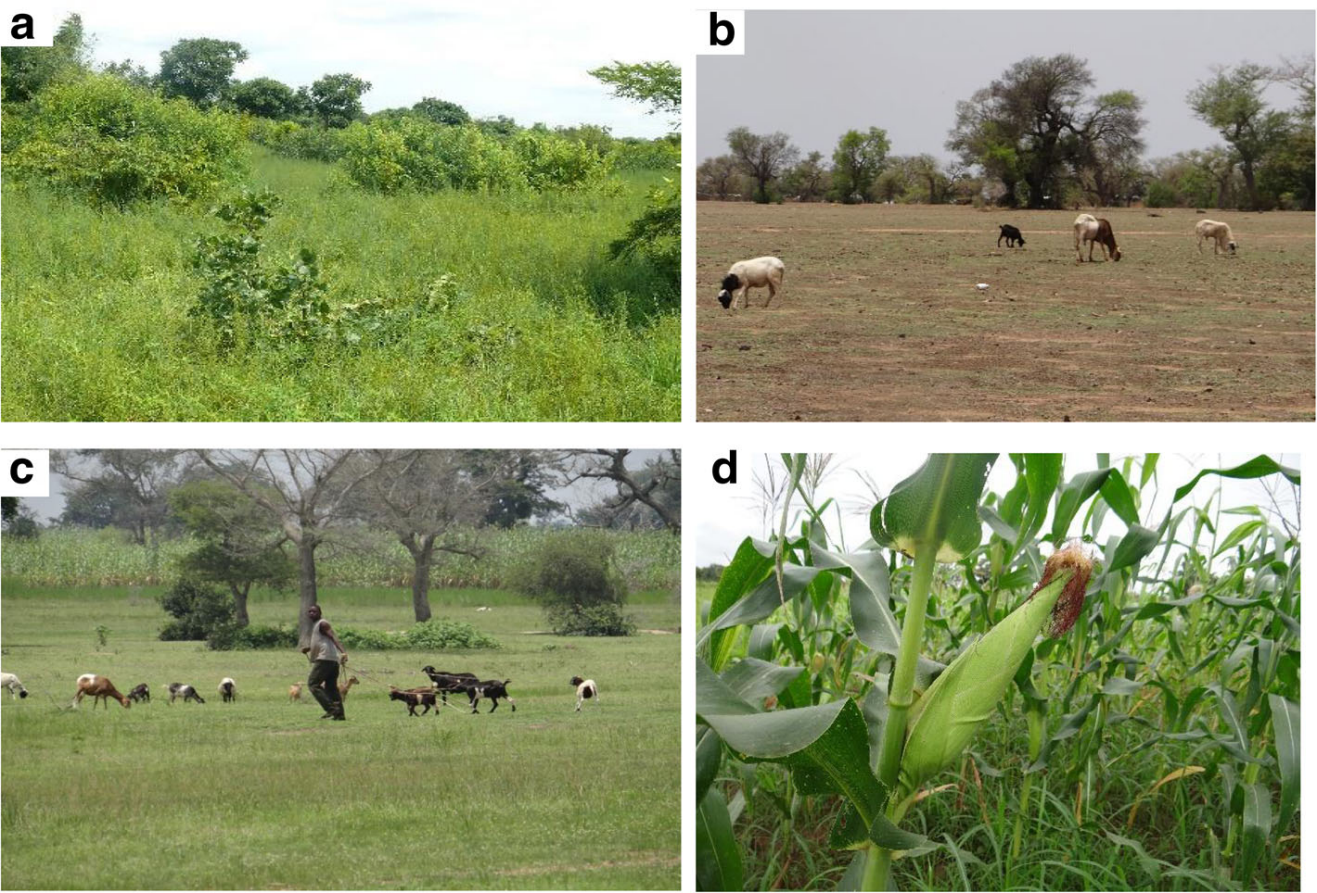
Local landscapes in the studied communities across both northern Ghana and southern Burkina Faso. **a** Bush (old fallow). **b** New fallow. **c** Reserved grazing field. **d** Cropland

The vegetation of the study area is characterized by open dry savanna type. Outside protected areas, the sparse tree layer mostly consists of economically important trees and shrubs such as the sheanut tree, *Vitellaria paradoxa*, or the baobab, *Adansonia digitata* [38, 39]. Some tree species which contribute to ruminant nutrition include *Afzelia africana*, *Pterocarpus erinaceus*, and *Piliostigma* spp. for cattle in particular while *Balanites aegyptiaca*, *Ziziphus mauritiana*, and *Acacia* spp. are primarily fed on by small ruminants [40]. The grass layer in the northern Sudanian pastures in Burkina Faso is dominated by *Andropogon pseudapricus*, *Loudetia togoensis*, *Aristida kerstingii*, *Dactyloctenium aegyptium*, and *Digitaria horizontalis* [40, 41]. The southern Sudanian zone is similarly dominated by *Andropogon* spp. while *Hyparrhenia* and *Schizachyrium* spp. are co-dominants in both northern Ghana and southern Burkina Faso [35, 41]. The singular distinguishing vegetation feature is that the northern Sudanian zone constitutes mostly patchy vegetation cover and many bare grounds [40] and fewer tree species, while the southern Sudanian zone has a continuous herbaceous cover interspersed with fire-resistant and broad-leaved trees [35]. The rangelands within which this study is conducted are mostly used for open communal grazing such as crop fields/farms, old and new fallows, and other arable lands (Fig. 2). There are no designated commercial rangelands in the studied communities for grazing purposes. Both herbaceous and woody species mentioned above are widespread within these rural communities. As mostly farmers, local agro-pastoralists cultivate all kinds of food and cash crops such as maize, groundnuts, guinea corn, millet, rice, and vegetables like tomatoes, okro, pepper, and other naturally available *Hibiscus* species in the bushes. Domesticated cattle, goats, and sheep are allowed to graze on these crop plants including their residues after the harvest period mostly in the dry season. Therefore, during the growing season, all small ruminants are tethered, and cattle are herded by cowboys to avoid destruction of crops on far away farmlands and compound farms around households.

### Cultural setting

This study largely focused on three dominant ethnic groups in the study area, namely Mossi in central and southern Burkina Faso, Gurunsi (with subethnic groups such as Frafra, Kasena, and Nabit) living on both sides of the border between Burkina Faso and Ghana, and Dagbani in northern Ghana (Fig. 1). These ethnicities share comparable agro-pastoral practices. Livestock husbandry constitutes a crucial aspect of the livelihood strategies of people living in rural semi-arid West Africa [42]. As agro-pastoralists, the smallholder farmers in the study areas engage in crop farming (monoculture or rotational crop farming) as well as in animal husbandry by providing natural grazing pasturage platforms to their livestock to cope with the unpredictable precipitation patterns. The dominant livestock types reared in the study areas include cattle, goats, and sheep (see Table 1 for vernacular names of these livestock types). Various crop residues are also considered to be vitally important for feeding their domestic livestock due to declining quality and quantity of available natural forage resources such as herbaceous plants [35, 41].

**Table 1:** Vernacular names for dominant livestock types (cattle, goats, and sheep) reared by local agro-pastoralists from different ethnic backgrounds in the study area

Livestock types	Dagbani vernacular	Mossi vernacular	Gurunsi vernacular		
			Frafra	Kasena	Nabit
Cow (Cattle)	*Naao* (*Nii*)	*Naafu* (*Niini*)	*Naaho* (*Nii*)	*Naao*(*Naani*)	*Nao*(*Nigi*)
Goat (Goats)	*Bua* (*Bue*)	*Buuga* (*Buusi*)	*Bua* (*Buusi*)	*Bugu* (*Bum*)	*Buo*(*Buus*)
Sheep (Sheep)	*Pegu* (*Peri*)	*Pisigu* (*Piisi*)	*Pisiku* (*Piisi*)	*Pie* (*Piini*)	*Piho*(*Pihi*)

### General sampling approach

To capture information on local valuation criteria for forage plants from local agro-pastoralists, a stratified random sampling based on important socio-demographic characteristics such as ethnicity, age, and gender was used. This stratification of local agro-pastoralists was replicated at each study site (village). The stratified random sampling was applied to collect representative data in the sampling population across ethnic groups, age classes, and gender affiliation. Five study villages per ethnic group were selected and further stratified per village by gender and age groups (Fig. 1). Age class definitions from previous studies in West Africa’s Sudanian savannas [15, 43] were used. Thus, local agro-pastoralists were distinguished into young (15–35 years), middle-aged (36–55 years), and old (> 55 years) adults. The ethnic and gender stratifications were also done based on dominant ethnic groups (e.g., Dagbani, Gurunsi, and Mossi) and males and females respectively [30]. Thus, the villages were generally nested within ethnic groups or aridity classes during the sampling process. This was done to disentangle the relative importance of these socio-demographic for valuation criteria among local agro-pastoralists in a consistent manner. Apart from the three main variables stated above, local agro-pastoralists’ residential status and educational backgrounds were also recorded. The majority (over 85%) of them are native residents while few migrant local agro-pastoralists are also resident in the research area. In sum, 526 local agro-pastoralists in 16 villages (seven in northern Ghana and nine in southern-central Burkina Faso) were covered, out of which at least 30 local agro-pastoralists were interviewed per village.

### Ethnoecological interviews

Prior to the commencement of the face-to-face interviews, the structured questionnaires were pre-tested with two local agro-pastoralists and fine-tuned so as to avoid too late questionnaire changes and to ensure easy understanding of research questions [30]. Considering the wide geographical spread and dialectical differences within the study area (Fig. 1), local field assistants from the three pre-determined ethnic groups (Dagbani, Gurunsi, and Mossi) were engaged to help translate research questions from English into respective local dialects to local agro-pastoralists. The respondents’ answers were then documented in English. Knowing that different local assistants may affect delivery of answers from local agro-pastoralists, their individual influences on answers given were minimized. This was done via adequate training of local assistants and pre-testing questions, and the author was personally present during interviews to ensure harmonization of the structured questions and associated answers given by local agro-pastoralists for documentation. Additionally, the different interpreters’ effects on answers were also reduced to the barest minimum by simplifying questions which needed straightforward answers. To gain local agro-pastoralists trust and their permission for the ethnobotanical surveys, the traditional chiefs and local authorities were contacted to ask for their permission and secondly proceeded to engage local agro-pastoralists whose consents were also sought prior to commencements of the individual-based interviews.

**Fig. 3:**
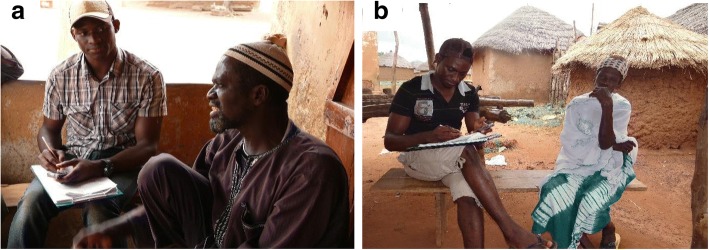
Author interviewing two local agro-pastoralists with the help of a local translator in northern Ghana in August 2013: **a** A male agro-pastoralist in Sang village and **b** a female agro-pastoralist in Nbatinga village

To better understand local agro-pastoralists’ local valuation criteria for forage plants on pastures, open-ended questions (free lists) were asked in the same manner during the ethnobotanical interviews for both male and female agro-pastoralists (Fig. 3). This provided local agro-pastoralists equal opportunity to answer a similar set of questions for subsequent comparison of responses and allowing them to express their knowledge and understanding on forage resource utilization in their own terms. This was similarly done by Bryman [44] and Kgosikoma et al. [45].

Following the free list tasks described by Naah et al. [30], local agro-pastoralists were asked to explicitly rank five forage plants (including crop plants/residues) from their free lists by starting from the most important to the least important forage plants or crop residues for their livestock (cattle, goats, and sheep). With respect to the seasonally variable importance of the free-listed forage plants, local agro-pastoralists were explicitly asked to separately cite and rank five of them particularly suitable for the dry season and the rainy season irrespective of livestock type. Regarding the local agro-pastoralists’ perceptions on palatability ranking of available forage plants, they were similarly asked to provide and rank five forage plants for each category of livestock. This was done because domestic ruminants have specific feeding preferences for available forage plants. In addition, local agro-pastoralists were also asked to list local plant species which are totally refused by their livestock. To better understand and appreciate why local agro-pastoralists explicitly ranked forage plants in terms of varied seasonal regimes and livestock-specific preferences, local agro-pastoralists were further asked to provide their own ranking criteria to allow for further content analysis of their responses.

### Climate data

The aridity stratification was done to determine whether climatic conditions did have a substantial effect on local forage valuation criteria provided by agro-pastoralists in a consistent manner. As done by Naah et al. [30], a climatic aridity value was calculated for each village using the UNEP aridity index, AI [46]. Thus, AI = P/PET, where P = mean annual precipitation and PET = annual potential evapotranspiration.

### Vegetation sampling strategy

Firstly, sample plots near to villages where the ethnobotanical interviews were conducted. To obtain ecological data on the tree layer, a standard Whittaker plot size of 20 m × 50 m [47] was used. Whittaker plots using different topographic gradient (upland, mid-slope, and lowland slopes) were established to maximize the homogeneity of the vegetation composition within the research area. Three Whittaker plots per each slope position were placed, totally nine plots per village. The relatively large plot size was chosen to take account of the patchy distribution of trees and shrubs and to capture most forage species at the sites.

For each Whittaker plot, a complete census of trees and shrubs was done, and all the tree/shrubby species were counted, identified and recorded. Thus, the stratified vegetation sample plots were in proximity to villages (plots not further than 10 km from villages) in which the predetermined dominant ethnic groups lived with the aim to avoid spatial autocorrelation. This sampling strategy was replicated in all 16 villages sampled, totaling 144 plots. This made it possible to subsequently match ethnobotanical and ecological datasets for better understanding the available forage resources utilization by local. For the local farmers, herbaceous species form a primary component of feed for their domestic livestock. Ideally, vegetation sampling on grasses should have been conducted. Nevertheless, this was not carried out, unlike the trees, for purely practical reasons such as large sample size, limited time and inadequate financial resources. Only woody component was considered in the vegetation sampling process because it was possible in the open savanna vegetation with scattered trees and shrubs. Additionally, it was interesting to find out which tree species were also suitable as forage source for feeding livestock since they have many competing uses (e.g., firewood, building materials, and shade provision, including forage) for the locals. Notwithstanding, this study is comprehensive and provided crucial information on available forage resources (grasses, trees, and crops) and their valuation by local agro-pastoralists.

### Voucher specimen preparation

Following the ethnobotanical interviews, substantial efforts were made to search and collect the cited forage plant species with the involvement of at least two knowledgeable local farmers from selected rural communities via ethnobotanical walks coupled with participant observation sessions [30, 48]. This was necessary because cited forage plant samples were only known in their vernacular names. For all 16 villages visited, about 64 ethnobotanical walks (averagely four walks per village) with two knowledgeable male local agro-pastoralists were done in each village to collect samples of forage species cited in local names for later scientific identification. A limited number of four participatory observation days were done to have first-hand observations on the kind of forage species grazed by cattle, goats and sheep in local landscapes. The collected forage plants (especially wild herbaceous and woody plants) were then herbarized according to standardized procedures such as labeling the specimens with a local name, date of collection, habitat/location of collection, and collector’s name. The taxonomic nomenclature of the herbarized forage plants was subsequently done at the University of Ouagadougou, Burkina Faso, via assistance from a well-trained technician and confirmed in the Senkenberg Institute in Germany. The taxonomic nomenclature of forage plants follows The Plant List [49]. As recommended by Nolan and Robbins, vernacular names obtained from the Mossi, Gurunsi, and Dagbani were cross-checked with already published vernaculars (e.g., [15, 17, 50–52]). The vernacular names given by indigenous people (in their own dialects) usually, reflect a wide spectrum of vital information on their understanding of such plants [53]. However, not all cited species were herbarized due to two reasons: (i) unavailability of cited forage species at the time of field research and (ii) taxonomic names were already known by the researchers especially crop plants and common food or economically important trees. A total of 558 citations of forage species given by local agro-pastoralists were only vernacularly known but could not be scientifically identified due to reason (i) given above.

### Data analysis

Reconciliation of vernacular and scientific names of forage species [28] was necessary because no scientifically pre-identified specimens of forage plants were used for the free list tasks. Thus, vernacular plant names given by local agro-pastoralists during the ethnobotanical interviews were subsequently matched to taxonomic names of such forage plants to establish their identities scientifically.

Also, descriptive statistics were conducted on the occurrence of groups of forage resources (e.g., trees/shrubs, herbaceous grasses/forbs, and crop-related forage plants) as explicitly ranked by local agro-pastoralists during the ethnobotanical interviews. Thus, the palatability of cited forage species was ranked based on seasonal differences (e.g., rainy and dry seasons) and livestock-specific preferences (e.g., cattle, goats, sheep). This was done with the aim to determine which forage resource types were mostly considered as most palatable for varied seasonal and livestock types in the study area. Forage species which were unidentified scientifically were excluded from further data analysis.

To quantitatively examine the salience of the explicitly ranked forage species in different local settings, the cognitive salience index (CSI) was calculated [54], which is based upon the frequency of forage species cited (F) and the mean position (mP) on free lists and sample size (N) for local agro-pastoralists. Thus, CSI = F/[N × mP]. The higher the CSI the higher the cultural importance (salience) of a forage species to local agro-pastoralists [55]. The CSI ranges from zero to one. The CSI values were calculated for each seasonal and livestock type considered in this study. Before detailed CSI analyses were carried out, forage species with no scientific information for only ethnobotanical-based data (CSI_ethno_) were excluded to ensure clarity in the explanation of the forage species salience results [28]. For all CSI-related analyses in this paper, ANTHROPAC 4.0 [56] statistical software was used.

Additionally, for the plot-based data or the ecological-based data (CSI_plot_) collected at various topographic positions in nearby local landscapes, where ethnobotanical interviews were done, forage species recorded per plot were equated to a free list of forage species provided by an individual respondent so as to apply the CSI formula above, as done by Linstädter et al. [28]. Thus, for the CSI_plot_ calculations on encountered forage species, CSI of a forage species was quantified as follows: F = the frequency of forage woody species as recorded on all sampled plots, mP = the mean position of recorded forage species as encountered on the plots, and N = the total number of plots sampled in all study sites, be it forage or non-forage species on the plots. With this statistical approach, it was possible to later link CSI_ethno_ and CSI_plot_ to establish any point of convergence with respect to forage plants availability, frequency, abundance, and salience.

In a similar vein, the “why” answers were treated as free listed items and the CSI values were calculated (as stated above) for valuation criteria for cited forage resources mentioned by local agro-pastoralists for livestock production and management, since they were asked to cite, as many as possible, their local valuation criteria for available forage resources. This innovative statistical approach provides information to quantitatively assess the salience of such valuation criteria or collective judgment of locally available forage plants for their livestock consumption and growth. In analogy to the CSI_ethno_ described above, F = the number of times a reason (valuation criteria) was mentioned by a local agro-pastoralist, mP = the mean position of a given reason by a local agro-pastoralist, and N = the total number of agro-pastoralists interviewed in the study region.

Furthermore, to examine the effects of socio-cultural and climatic variables on the citation of explicit valuation criteria of forage resources by local agro-pastoralists, a series of generalized linear mixed-effects models (GLMM) was performed with a Poisson error distribution and a (log) likelihood-based model selection procedure [30, 57], eliminating non-significant effect or interaction-terms [58]. Thus, the number of different kinds of valuation criteria for rainy season (Cri_RS_), dry season (Cri_DS_), cattle (Cri_cattle_), goats (Cri_goats_), and sheep (Cri_sheep_) cited by local agro-pastoralists was treated as count response variables, while the socio-cultural and environmental settings as predictor variables (ethnicity, aridity class, age class, gender, educational level, and residential status), representing the fixed-effect terms. It is important to note that age class and educational level were modeled as ordered factors while ethnicity, aridity class, gender, and residential status were used as just factors in the model selection process. Also, aridity class was used as a categorical variable instead of using it as a continuous variable in the model selection process because of easy comparison of such results to that of ethnic group as a categorical variable. Moreover, in modeling aridity class as a continuous variable, the results were not significantly different from that of the aridity class. The GLMM approach was used because of the count response variable (number of criteria mentioned per local agro-pastoralist).

From a correlation-matrix obtained using principal components analysis (PCA) analysis on the predictor variables considered for this study with varimax rotation, the ethnicity and climatic aridity variables were found to be collinear. Two separate initial global models (ethnicity-based and aridity-based models) were established. They only differ in terms of inclusion of either of these terms, for each Cri_RS_, Cri_DS_, Cri_cattle_, Cri_goats_, and Cri_sheep_, totaling ten competing models. This was done to assess the relative importance of ethnicity and aridity variables in determining the valuation of forage resources by local agro-pastoralists. As a nested design in this stratified study, whereby villages/sites were nested within either ethnic groups or aridity classes, 1|Village/aridity class or ethnic group was used as a random (intercept) term. This was done to account for potential site-specific differences [58]. This then means that *p* values in the results only reflect the main effects or interacting effects of the fixed-terms but not considering the possible effect of site/village on differences in the local valuation criteria for forage resources in the GLMM approach used.

For the final models in all cases, Akaike Information Criterion (AIC) values were evaluated. The most parsimonious model was then selected as the final model, following the principle of parsimony [59] and performed further analyses on the aridity-based models (Table 4). The finals models were subsequently analyzed by using ANOVAs (type III) and Turkey contrasts to determine multiple comparisons of means. The marginal, *R*_m_^2^ (variance explained by only the fixed terms), and conditional, *R*_C_^2^ (variance explained by both fixed- and random-terms), in the responses were calculated. Statistical assumptions were graphically checked by plotting residuals to check normality of errors and homogeneity of variance [60]. In the data analysis, the problem of over-dispersion (where the variance is greater than the mean) was not encountered. The model selection procedures, ANOVAs analysis, and *R*^2^ were performed with the lme4 and *R*^2^_GLMM (best)_ packages in R statistical software v.3.2.0 [61], while the exploratory analyses were carried out with IBM SPSS version 23 [62].

## Results and discussion

### Seasonal- and livestock-specific rankings and salience of forage species among local agro-pastoralists

**Fig. 4:**
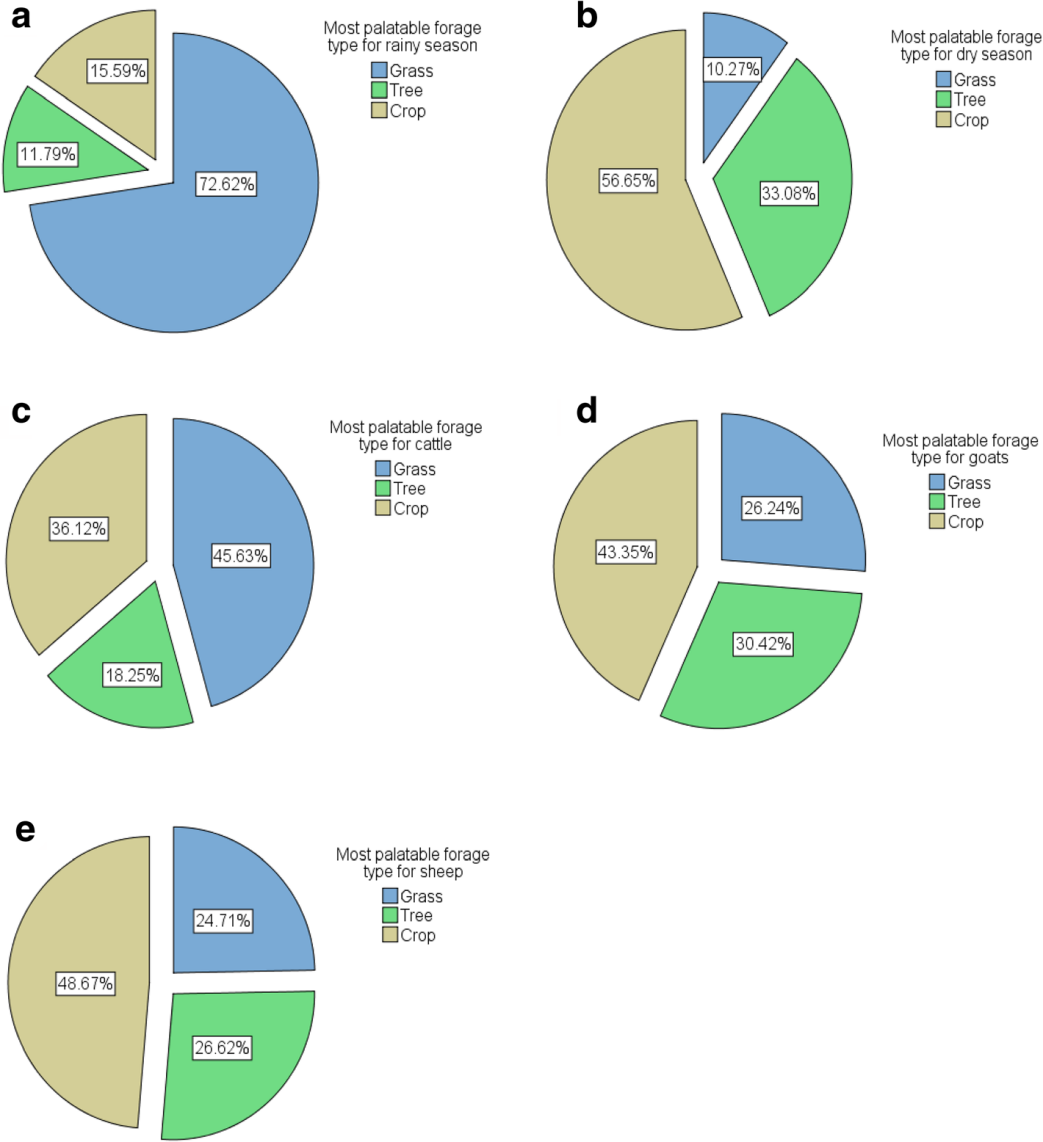
**a**–**e** Proportions of forage plants types ranked by local farmers as most palatable or suitable during rainy and dry seasons and for cattle, goats, and sheep production respectively

Local agro-pastoral SESs stem from the daily utilization of various forage resource types used by dominant domestic livestock (e.g., cattle, goats, and sheep) over the years within the study areas. When asked to specifically rank forage-related resources for different seasonal and livestock types, the local agro-pastoralists (belonging to the Dagbani, Gurunsi, and Mossi ethic groups) expressed their local knowledge in a wide range of various forage plants in their answers (see Table 2 for various forage-related vernacular names for cover terms). Based on the seasonal ranking, the findings revealed that 73% of local agro-pastoralists ranked herbaceous plants (grasses and forbs) as most palatable for feeding domestic livestock in the rainy season as compared to 27% of them who ranked crops (fresh crops/crop residues) and woody vegetation (trees and shrubs leaves) as most palatable for their livestock in the same season (Fig. 4a). This may be explained by the fact that grasses and forbs are fresher, more nutritious and highly digestible at their early phenological growth stage for livestock consumption in the rainy season [63]. It may also be due to herbaceous plants being considered as primary food sources for livestock consumption as well as being more abundant forage plants in the rainy season. Thus, there may be no urgent need for animals themselves or livestock owners to look for supplementary feeds such as crop-related forage and leaves of trees and shrubs in this season. It is thus evident that savanna grassland ecosystems are very important for livestock. Nonetheless for the dry season, approximately 57% and 33% of local agro-pastoralists highly ranked crops and woody vegetation respectively (compared to 10% for the herbaceous forage plants; Fig. 4b). This may be largely attributable to scarcity or unavailability of good herbaceous forage plants, on the one hand, and tree leaves and crop residues are readily available in the harsher dry season on the other hand. Woody species are not only important for domestic livestock but also for the owners themselves since leaves of trees are used as animal feed and cooked as sauce for people, as was reported by Krohmer [19] among the Sahelian Fulani in northern Burkina Faso. This is also true for the herbaceous and crop-related forage plants. A similar study done in the semi-arid region of northwestern Brazil by Nunes et al. [48] also reported that local agro-pastoralists cited more herbaceous forage species for the rainy season than for the dry season while vice versa was true for the citation of woody forage species by local agro-pastoralists, indicating how climatic factors modulate forage quality and quantity. The local agro-pastoralists exhibited deep understanding and perception on the dynamics of forage value which enable them to provide alternative feeding materials for their livestock in the face variable precipitation patterns. This is because they know exactly what forage resource type is preferable for a livestock type and at a particular season as demonstrated in the results above. The results also suggest that crop residues were much more preferred by domestic livestock than woody vegetation in the lean (dry) season (Fig. 4b). As indicated by Waziri et al. [64], having knowledge in various constituents of livestock feed is pivotal to production and productivity. About 60% of livestock feeds that are provided by rural population come from crops and crop residues (J. B. Walier, Head of Crop Division, MOFA, Bolgatanga-Personal communication).

**Table 2:** Vernacular names for cover terms of various forage types used by cattle, goats and sheep given by local agro-pastoralists from different ethnic backgrounds in the study areas

Forage types	Dagbani vernacular	Mossi vernacular	Gurunsi vernacular		
			Frafra	Kasena	Nabit
Grasse(s)	*Mogu* (*More*)	*Moo* (*Moogu*)	*Muo/mooro*	*Gaa* (*Gao*)	*Muo*(*Muut*)
Tree(s)	*Tia* (*Tiihi*)	*Tiiga* (*Tiisi*)	*Tia/Tiisi*	*Teo* (*Teeni*)	*Tii*(*Tiih*)
Crop(s)	*Binderogu*	*Yambri* (*Yamdo*)	*Buu/Buusi*	*Varawudeo*(*-diiro*)	*Zoot*(*Zoot*)

Regarding livestock-specific preferences, the results revealed that the preferential rankings for cited forage resource types by local agro-pastoralists were different for targeted livestock types irrespective of the seasonal type. Local agro-pastoralists reported that cattle liked herbaceous forage plants more than goats and sheep (Fig. 4c). Conversely, goats and sheep liked crop-related forage plants more than cattle (Fig. 4d, e). Similarly, this is true for woody vegetation. Goats and sheep tend to prefer tree, and shrub leaves as good feed sources more than cattle (Fig. 4d, e). These findings do support existing scientific literature [65] in that, cattle are mainly described as grazers, goats are generally browsers, and sheep are considered as intermediate feeders. The different preferences of these commonly raised livestock types for herbaceous composition, woody vegetation, and crop-related forage plants can be used to increase forage utilization and efficiency, suggesting the importance of integrated feeding mechanism usually employed by local land users. In current times of unpredictable weather conditions in the study region, forage-related LEK is required for sustainable livestock production and management as crop cultivation is sensitive to rainfall variability, leading to low crop yields for farmers.

**Fig. 5:**
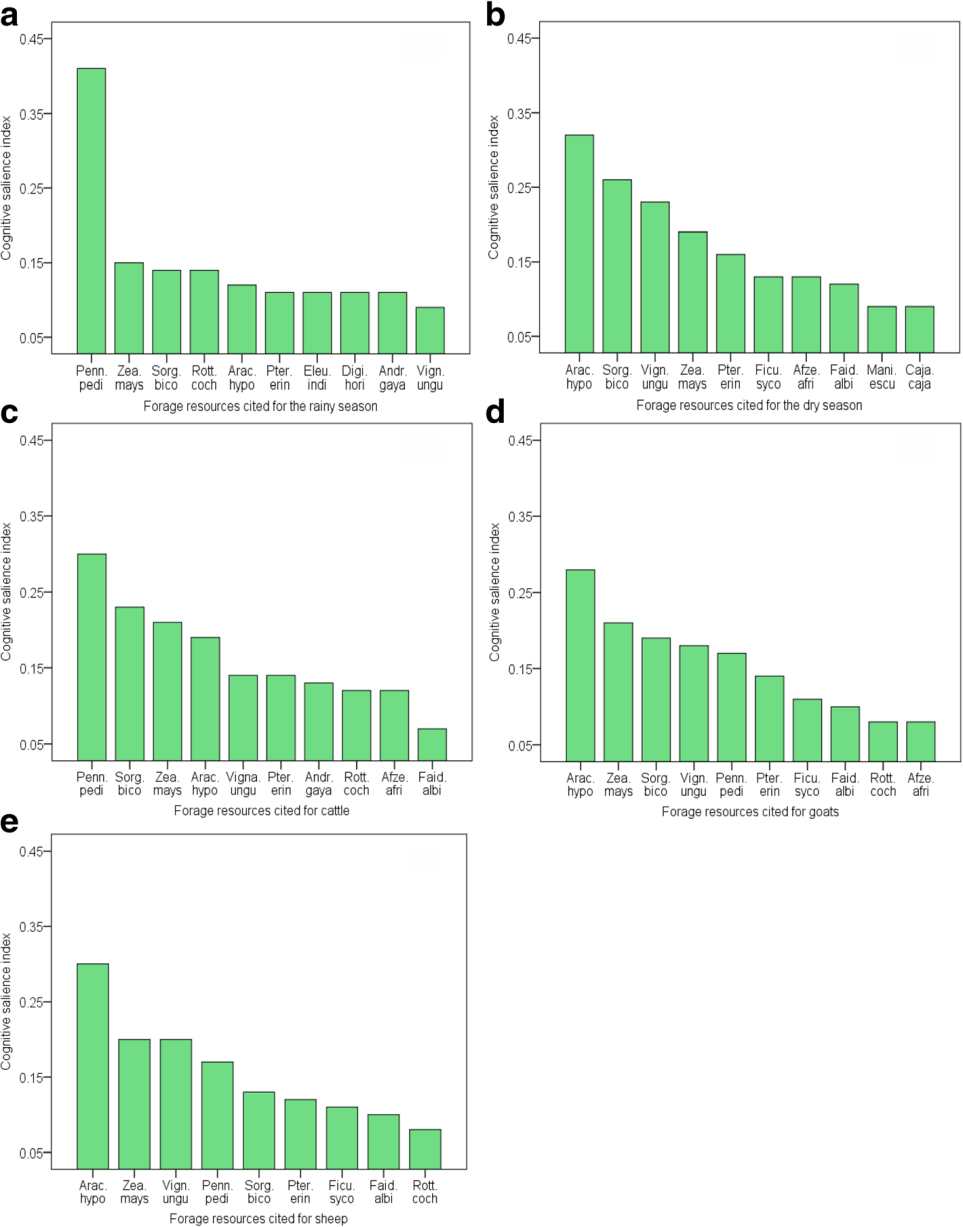
**a**–**e** Cognitive salience indices of the 10 most commonly cited individual forage species by agro-pastoralists in descending order in 16 villages located in northern Ghana and southern Burkina Faso. Note: Penn.pedi = *Pennisetum pedicellatum* Trin, Zea.mays = *Zea mays* L., Sorg.bico = *Sorghum bicolor* (L.) Moench, Rott.coch = *Rottboellia cochinchinensis* (Lour.) W. D. Clayton, Arac.hypo = *Arachis hypogaea* L., Eleu.indi = *Eleusine indica* (L.) Gaertn, Andr.gaya = *Andropogon gayanus* Kunth, Pter.erin = *Pterocarpus erinaceus* Lam., Digi.hori = *Digitaria horizontalis* Willdenow, Vign.ungu = *Vigna unguiculata* (L.) Walp, Ficu.syco = *Ficus sycomorus* L., Afze.afri = *Afzelia africana* Smith ex Pers., Faid.albi = *Faidherbia albida* (Del.), Mani.escu = *Manihot esculenta* Crantz and Caja.caja = *Cajanus cajan* (L.) Millsp

In calculating the cognitive salience indices (CSIs) of individual forage species ranked by local agro-pastoralists across all climatic aridity classes (moist semi-arid, dry semi-arid, dry sub-humid, and humid) covered in this study, *Pennisetum pedicellatum* Trin was adjudged the most salient herbaceous species with CSI values of 41% and 30% for the rainy season and cattle respectively among the top 10 forage species ranked (Fig. 5a, b). Also, *Arachis hypogaea* L. was ranked as the topmost forage species with corresponding CSI values of 32% for the dry season and 28% and 30% for goats and sheep respectively as compared to other highly ranked forage species for these different targeted seasonal and livestock types (Fig. 5c–e). The CSI values reflect the collective cultural importance of highly ranked forage plants to the local agro-pastoralists, since these forage species form an integral part of their livestock feed source. Various studies have shown that *P. pedicellatum* Trin and *A. hypogaea* L. have been found to have very high nutritional quality such as high crude protein, crude fiber, ash content, calcium/carbohydrates, fatty acid, amino acid, and in vitro digestibility profiles [64, 66]. This may make them most suitable to livestock and in turn highly ranked by local farmers. As an annual, *P. pedicellatum* Trin tends to grow faster during the growing (rainy) season and has more abundant leaves compared to *Andropogon gayanus* Kunth [64]. Additionally, *P. pedicellatum* Trin and *A. hypogaea* L. might have been highly ranked and that may be due to the widespread presence of the former and growing of the latter on farmlands by local agro-pastoralists in the studied communities.

Regarding culturally important woody species in the study area, the ethnobotanical surveys revealed that *Pterocarpus erinaceus* Lam, *Ficus sycomorus* L., and *Afzelia Africana* Smith ex Pers were well recalled by local agro-pastoralists to have very good forage value for livestock. This finding, especially on *P. erinaceus* Lam and *A. Africana* Smith ex Pers, was corroborated by an ethnobotanical study on biodiversity conservation of useful woody species in neighboring Benin’s Wari-Maro forest reserve [67]. However, these useful woody species were much less represented (fewer in number) in many villages in both Ghana and Burkina Faso. This is because the numbers of these woody forage species were confirmed by the vegetation sampling data since they were less recorded or completely missing in some plots. It was rather *Vitelliara paradoxa* C. F. Gaertn, *Piliostigma reticulatum* (DC.) Hochst, and *Lannea microc*arpa Engl. & K. Krause which were commonly found and dominated in the plot-based dataset but were least cited by local agro-pastoralists as good forage resources for their livestock. These results could be explained by the fact that some species including economically useful trees may be present in the local environment but not necessarily be considered as favorable forage resources for sustainable livestock production by local agro-pastoralists. The dominance of the Poaceae and Fabaceae families from which many forage species come do reflect their high forage potential, as reported in several ethnobotanical studies in the region [17, 30] and elsewhere [48, 68]. The vast local knowledge of agro-pastoralists on forage resources and their valuation is reflected in their answers in the context of (agro-)pastoral ecosystem. Despite the constraints of access to forage resources to domestic livestock, pastoralists and/or agro-pastoralists play a key role in determining foraging patterns by assisting grazing animals to accomplish optimal foraging behavior [25].

### Salience of explicit criteria for valuating forage resources for livestock production and management among agro-pastoralists

The results indicated that local agro-pastoralists judged individual forage plants for various livestock and seasonal types based on a myriad of reasons. The findings of this study support the hypothesis that the local valuation of forage resources is based on several criteria during different seasons and for different livestock types. Among many other reasons, the CSI results revealed that “healthy growth of livestock” was consistently regarded as the most culturally important underlining criterion for ranking forage resources for different seasons (e.g., rainy and dry seasons) and livestock types (e.g., cattle, goats, and sheep). The CSI values were consistently highest for the healthy growth of livestock criterion ranged from 27 to 44% under all seasonal and livestock types (Table 3). This criterion is highly important to local agro-pastoralists since healthy animals have direct financial benefit for them because they can sell their livestock at good prices on local markets. Therefore, forage resources which were able to ensure healthy growth of their livestock were ranked highly among other ones. Also, depending on the season, local agro-pastoralists stated the availability of forage resources as a criterion for preferential ranging. For instance, “availability of grasses” was a very important criterion given for the rainy season with CSI value of 19% while “availability of crops and trees” and “unavailability of fresh grasses” criteria were regarded vitally important for the dry season with same CSI value of 13% (Table 3). This is not surprising because fresh grasses and forbs are readily available and adjudged by local agro-pastoralists as very important for grazing purposes in the rainy season. Other important criterion cited for ranking local forage plants was related to their ability to make local farmers’ livestock “grow fat” as shown with CSI value of 10% (Table 3). This then accounts for other valuation criteria cited such as “milk production” and “taste”. In the dry season, herbaceous forage plants are largely old and matured or may be burnt by bushfires and they are thus less important to livestock grazing. It is rather forage-related crops and leaves of trees that are available and more important for domestic livestock during the dry season. This is because crops/crop residues and leaves of trees/shrubs which were very palatable for feeding livestock in the dry season. Additionally, the perceived livestock desires in both seasons, and forage palatability at a young phonological stage were very strong reasons for ranking of forage species particularly for the rainy season (Table 3). The reason of livestock growing fat after feeding on a forage plant as well as their “taste” was very salient criteria for cattle (Table 3). This may be explained by local agro-pastoralists’ intention to sell cattle with fine skins and fat body conditions at high prices or to use bullocks for plowing purposes. For goats and sheep, similar criteria of “healthy growth of livestock,” “grow fat,” and “taste” formed the basis for the preferential ranking of forage plants by local agro-pastoralists. Other criteria for valuation of forage resources for cattle, goats, and sheep include “milk production,” “hunger,” “increased reproduction,” and “nutrients (vitamins)” as well as general reason such as “natural food source,” “good for our animals,” and “energy provision” for livestock survival (Table 3). A similar study on the use of local fodder flora in Pakistan also reported that woody vegetation (especially *Acacia nilotica* (L.) Willd. ex Delile and *Ziziphus mauritiana* Lam.) was the most preferred forage species for goats and camel but not cattle and sheep due to their ability to satisfy, ever green nature and sweetness [69]. However, this study failed to quantify the salience of these examples of criteria given by local farmers, unlike this study. Duku et al. [70] stated that smallholder farmers ranked their feed sources for small ruminants based on a multiplicity of reasons such as their availability, palatability, proximity, abundance, reliability and health risks in the transitional zone of Ghana. However, this study and others failed to quantify the salience of valuation criteria of locally available forage species.

**Table 3:** The topmost 15 local valuation criteria provided by local agro-pastoralists and their respective cognitive salience indices (CSIs) for rainy season, dry season, cattle, goats, and sheep. The CSI values which are more than 10% of 10 topmost valuation criteria mentioned per each case (rainy season, dry season, cattle, goats, and sheep) are in italic figures

Local valuation criteria	Salience RS	Salience DS	Salience cattle	Salience goats	Salience sheep
Healthy growth of livestock	*0.347*	*0.267*	*0.432*	*0.426*	*0.435*
Availability of grasses	*0.189*				
Animal desires	*0.154*	0.013			
Phenological stage of grasses	*0.118*				
Grow fat	*0.104*	0.078	*0.123*	*0.115*	*0.117*
Hunger	0.086	0.080	0.094	*0.106*	*0.102*
Energy provision	0.072	0.052	0.074	0.072	0.062
Natural food source	0.069	0.027	0.069	0.093	0.084
Taste	0.042	0.033	*0.111*	*0.108*	*0.101*
Milk production	0.024	0.02	0.032	0.027	0.030
Availability of crops and trees		*0.133*			
Unavailability of fresh grasses		*0.127*			
Nutrient (vitamins)			0.038	0.038	*0.100*
Good for our animals			0.034	0.034	0.034
Increased reproduction			0.021	0.027	0.016

### Determinants of citation of explicit valuation criteria for forage resources by local agro-pastoralists

Based on the AIC values obtained from established candidate global models, the aridity-based models were retained in the “best” final models for all explanatory variables namely criteria for rainy season (Cri_RS_), dry season (Cri_DS_), cattle (Cri_cattle_), goats (Cri_goats_), and sheep (Cri_sheep_) considered. This finding is not surprising because both aridity and ethnicity variables were found to be collinear since both variables were having similar dimension in terms of coverage in the study area (Fig. 1). The retained aridity-based models were subjected to further analysis and discussion in this paper, since aridity class variable seems to significantly contribute to the variance explained in the citation of valuation criteria for forage plants by local agro-pastoralists as compared to the less influential ethnicity-based models. It was revealed that the main effects of aridity classes but not the interacting effects of it and gender, age, educational and residential status variables of the local agro-pastoralists were found to be significant. The compared delta AIC values of aridity- and ethnicity-based final models were found to be plausible since it was greater than two, as was similarly reported by Naah et al. [30]. The results also revealed that climatic aridity had a strongly significant effect on the number of citation of forage-related valuation criteria necessary for livestock production among local agro-pastoralists during the rainy season (Cri_RS_; χ^2^ = 70.17, Df = 3, *p* = < 0.001, Table 4), dry season (Cri_DS_; χ^2^ = 107.17, Df = 3, *p* = < 0.001, Table 4), for cattle (Cri_cattle_; χ^2^ = 58.92, *p* = < 0.001), goats (Cri_goats_; χ^2^ = 62.39, *p* = < 0.001, Table 4), and sheep (Cri_sheep_; χ^2^ = 74.95, Df = 3, *p* = < 0.001, Table 4). For all valuation criteria cited for dry and wet seasons, cattle, goats, and sheep, agro-pastoralists living in humid and dry sub-humid locations gave many different reasons for ranking their forage plants while those in semi-arid villages cited fewer reasons. Pairwise comparisons with adjusted *p* values showed that there were significant differences with respect to Cri_RS_ in moist semi-arid (MSA) and dry semi-arid (DSA) locations (*p* = < 0.001, *r* (effect size) = 0.26). This was similarly observed between Cri_RS_ in dry sub-humid (DSH) and DSA (*p* = 0.001, *r* = 0.33), humid (HUM) and DSA (*p* = 0.001, *r* = 0.30). There was, however, no significant effect of aridity on Cri_RS_ when compared between DSH and MSA, HUM and MSA, and HUM and DSH locations (*p*s > 0.05). Comparing the Cri_DS_ between MSA and HUM localities, the follow-up post hoc tests revealed that local agro-pastoralists living in the former rather cited significantly fewer Cri_DS_ than those in the latter location (*p* = < 0.001, *r* = 0.18). A similar significant difference was found between MSA and DSH (*p* = < 0.001, *r* = 0.17) villages as opposed to the same aridity classes in the Cri_DS_ as explained above. The Cri_DS_ was found to be significantly cited by agro-pastoralists living in MSA, DSH, and HUM as compared to those residing in DSA areas (*p* = 0.001). Pairwise comparisons for Cri_cattle_ and Cri_goats_ showed local agro-pastoralists inhabiting DSH, HUM, and MSA environments cited a significantly higher number of local valuation criteria for suitable forage plants for cattle and goats’ consumption than that of the DSA areas (*p*s = < 0.001). For the Cri_sheep_, a similar pattern of local ecological knowledge was found as explained for Cri_cattle_ and Cri_goats_ above. Naah et al. [30] similarly found a significantly higher level of LEK on forage plants among local agro-pastoralists in wet environmental conditions than those in dry landscapes. The way in which the differences exist in various aridity classes, as illustrated above, is a testament of how varying climatic conditions encourage local agro-pastoralists to have many more reasons for many forage species cited in humid areas as compared to fewer reasons for fewer forage species mentioned in drier areas, as such weather conditions directly affect forage species availability and distribution. This may also be attributable to local agro-pastoralists in wet vegetation areas being willing to brainstorm various kinds of their valuation criteria as compared to those in arid parts of the study region whose major interest may be the healthy upkeep of their livestock partly due to limited availability of forage plants. Studies have showed that climatic aridity or harshness is a major driver of change to rain-fed crop cultivation and to some extend livestock production [7] and one of the most important environmental variables for forage provision and erosion control in West African savannas [31] and similarly reported elsewhere [71]. Some authors have argued that when they directly asked local farmers’ perceptions on climate change and agricultural adaptation strategies, climate is found to have a limited direct defining role [72, 73]. Grazing pressure by domestic livestock leading to overgrazing is observed to be existing in some grazed areas of the study region, especially on the drier side of Burkina Faso, which can compound the negative effects of climatic aridity. It is therefore recommended that specific climate change impacts studies should be undertaken to confirm climate influence on citation of valuation criteria of natural forage resources management by local agro-pastoralists in the study region. The results of this ethnoecological study may contribute to sustainable management of forage resources and farmers’ livelihoods as their local knowledge has far-reaching positive effects on younger generations in these local communities. The *R*_m_^2^ and *R*_c_^2^ calculated were rather found to be generally low. Depending upon the explanatory variable considered, the *R*_m_^2^ ranges from 21 to 25% while *R*_c_^2^ ranges from 23 to 26% of variance explained in the responses (Table 4).

**Table 4:** Results of testing fixed-effects of aridity class variable using generalized linear mixed-effects (GLMM) on number of local valuation criteria cited by agro-pastoralists for (I) rainy season (Cri_RS_), (II) dry season (Cri_DS_) and (III) cattle (Cri_cattle_), while aridity class and educational background of local agro-pastoralists influenced (IV) goats (Cri_goats_) and (V) sheep (Cri_sheep_) as metric of regional-level variance. Detailed corresponding follow-up post hoc tests for the analysis of deviance results using Wald chi-square (χ^2^) tests were also calculated

Valuation criteria	Cri_RS_	Cri_DS_	Cri_cattle_	Cri_goats_	Cri_sheep_
(Intercept), χ^2^	13.00	8.61	6.94	8.19	9.32
Aridity class, χ^2^	70.17	107.17	58.92	62.39	74.95
Marginal, *R*_m_^2^ (%)	21	23	23	24	25
Conditional, *R*_c_^2^ (%)	23	23	26	26	26
*p*	***	***	***	***	***

Random effect = ~ 1|Village/Aridity class (village nested within aridity class variable to account for site-specific variations)

*DSA* dry semi-arid, *MSA* moist semi-arid, *DSH* dry sub-humid, *HUM* humid

p* = < 0.05, **p = < 0.01, ***p = < 0.001; Df = 1 for “Intercept” and 3 for “Aridity class” in each criterion case

## Conclusions

This study has provided an account of forage resource types and valuation criteria from local agro-pastoralists’ perspectives along a steep climatic gradient in West African savanna vegetation (covering northern Ghana and southern-central Burkina Faso). The results of this research revealed that local agro-pastoralists exhibited extensive knowledge in various forage resource types (e.g., herbaceous, woody vegetation, and crop residues) used by domestic livestock (e.g., cattle, goats, and sheep) at different seasons (e.g., dry and rainy seasons). The results further indicated that herbaceous forage species were ranked as most palatable for feeding domestic livestock in the rainy season while forage-related crops and woody vegetation were highly ranked for livestock grazing in the dry season. It is important to note that valuation criteria with high CSI values for preferential rankings of forage species are of great cultural importance and popularity among local agro-pastoralists for humid and semi-arid areas. The results also suggest the importance of savanna grassland ecosystems to provide various forage sources for sustainable livestock production. Climatic aridity has a significant effect on how forage resources are adjudged by local agro-pastoralists. Thus, local people in humid and sub-dry humid villages generally provided many reasons for their ranked forage plants as compared to those living in moist and dry semi-arid localities. This also affirms the fact that local resource-users do not just behave in a “vacuum” but consciously make their choices on the use of such limiting forage resources based on underlying reasons and prevailing circumstances for sustained livestock production and livelihood improvement. Knowing which forage types are suitable for various livestock and seasonal types enables local agro-pastoralists to better plan and manage available forage resources in the face of changing local climatic conditions in a sustainable manner. This approach may help us appreciate how local land users perceive and utilize their forage resources in both periods of abundance and scarcity. This is because management-related decisions taken on the utilization of declining forage resources by agro-pastoralists at the local level is extremely crucial for understanding global climate change dynamics on conservation of forage species for future generations. Literature has shown that the role of development interventions for increasing adaptive capacity is vitally important for understanding the relationship between poverty and vulnerability, which will in turn inform policy decisions globally [74]. In conclusion, this study highlights the continued importance of local ecological knowledge for natural resource management. It is thus recommended that a lot more attention should be given to LEK-related investigations in dryland ecosystems to ensure sustainable use of forage plants for improved livestock production and also stimulate the scholarly debate about the resilience of local agro-pastoral SESs for effective natural resources management.

## Abbreviations

AI: Aridity index
AIC: Akaike Information Criterion
Cri_cattle_: Valuation criteria for cattle
Cri_DS_: Valuation criteria for dry season
Cri_goats_: Valuation criteria for goats
Cri_RS_: Valuation criteria for rainy season
Cri_sheep_: Valuation criteria for sheep cited by the local agro-pastoralists
CSI: Cognitive salience index
CSI_ethno_: Ethnobotanical-based data
CSI_plot_: Plot-based data or the ecological-based data
DSA: Dry semi-arid
DSH: Dry sub-humid
GLMM: Generalized linear mixed-effects models
HUM: Humid locations
LEK: Local ecological knowledge
MAP: Mean annual precipitation
MSA: Moist semi-arid
PCA: Principal components analysis
PET: Annual potential evapotranspiration
SES: Social-ecological systems
WASS: West African Sub-Sahara
